# Ultra-Thin Porous PDLLA Films Promote Generation, Maintenance, and Viability of Stem Cell Spheroids

**DOI:** 10.3389/fbioe.2021.674384

**Published:** 2021-06-14

**Authors:** Ya An Tsai, Tianshu Li, Lucia A. Torres-Fernández, Stefan C. Weise, Waldemar Kolanus, Shinji Takeoka

**Affiliations:** ^1^Department of Life Science and Medical Bioscience, Graduate School of Advanced Science and Engineering, Waseda University (TWIns), Tokyo, Japan; ^2^Institute for Advanced Research of Biosystem Dynamics, Research Institute for Science and Engineering, Waseda University, Tokyo, Japan; ^3^Life and Medical Sciences Institute (LIMES), University of Bonn, Bonn, Germany

**Keywords:** ultra-thin film, porous nanosheet, PDLLA, 3D spheroid culture, cell viability, cell division

## Abstract

Three-dimensional (3D) culture bridges and minimizes the gap between *in vitro* and *in vivo* states of cells and various 3D culture systems have been developed according to different approaches. However, most of these approaches are either complicated to operate, or costive to scale up. Therefore, a simple method for stem cell spheroid formation and preservation was proposed using poly(D,L-lactic acid) porous thin film (porous nanosheet), which were fabricated by a roll-to-roll gravure coating method combining a solvent etching process. The obtained porous nanosheet was less than 200 nm in thickness and had an average pore area of 6.6 μm^2^ with a porosity of 0.887. It offered a semi-adhesive surface for stem cells to form spheroids and maintained the average spheroid diameter below 100 μm for 5 days. In comparison to the spheroids formed in suspension culture, the porous nanosheets improved cell viability and cell division rate, suggesting the better feasibility to be applied as 3D culture scaffolds.

## Introduction

Two-dimensional (2D) cell culture on tissue plates is commonly performed in *in vitro* cell biology studies, allowing researchers to study how cells expand, behave under various stresses, proliferate and/or differentiate in response to stimuli. Monolayer incubation, however, lacks fundamental cell-cell and cell-matrix interactions as well as direct cell-to-cell exchange of chemical cues, thus limiting our understanding on how cells behave *in vivo*. Compared to 2D cell culture, three-dimensional (3D) culture bridges the gap between cell physiology and *in vitro* cell culture and improves *in vitro* to close-to-*in vivo* research. It provides more practical tool in basic and applied research to broaden the perspective of cell biology.

3D cell aggregates (spheroids) generate an inherent nutrient and oxygen gradient within their structure and constitute an *in vivo* microenvironment. Previous studies using 3D arranged cells uncovered mechanisms involved in tumorigenesis (Lv et al., 2017) and in maintaining expression of specific biomarkers due to enhanced extracellular matrix (ECM) (Bazou, 2010), and demonstrated the ability of spheroids to serve for drug evaluation and development. Stem cell spheroids are especially promising to improve the therapeutic effects of stem cell therapy through raised cell viability, cell secretomes and differentiation ability (Park et al., 2017). Embryonic stem cell spheroids have shown multiple functional capabilities in development and differentiation (McKee and Chaudhry, 2017). In addition, mesenchymal stem cells cultured in 3D systems exhibit secretion of anti-inflammatory factors (Bartosh et al., 2010), enhanced cell survival (Emmert et al., 2013) and the osteogenic differentiation ability for bone regeneration application (Yamaguchi et al., 2014).

3D cultures made in different culture environments aid in our understanding of cell physiology changes by cell morphology observation, cytoskeleton examination, and cell-cell junction visualization within aggregates. Numerous techniques have been published for 3D cell cultures and classified according to the methodology of scaffold-free or scaffold-based systems. Scaffold-free techniques based on non- or limited support provide low-cost, simple and rapid cell aggregation via gravitation from the hanging drop and pellet culture methods (Santos et al., 2015; Xu et al., 2016) to form spheroids. These spheroids are easy to harvest but difficult to expand in a continuous passage (Lv et al., 2017). On the other hand, scaffold-based systems could construct a culture environment that is much closer to the *in vivo* conditions. For example, chitosan-based scaffolds or gel-supported embeddings chemically interact with cell spheroids, provide dense ECM signal connections and induce focal adhesion protein expressions (Huang et al., 2011; Zujur et al., 2017). However, these techniques require relatively complicated chemical reactions (Kim et al., 2013) and are costly in large-scale production (Lv et al., 2017). Therefore, economic biomaterials and easy-to-handle procedures are desired to achieve large-scale 3D cultures.

We have developed nanosheets with biocompatible and biodegradable organic polymers which are free-standing thin films, with tens or hundreds of nanometers in thinness (Fujie, 2016). They are suitable to be applied in a variety of biomedical applications such as an alternative to bio-membranes, for drug loading or as wound dressing materials (Fujie, 2016). They were also applied as wrapping materials to achieve high-quality live imaging of tissue and suspension cells (Zhang et al., 2017, 2018).

The tissue-biomaterial interface is pivotal in regulating the cellular interactions, for example cell adhesion, morphology, orientation, motility, proliferation, and differentiation as well as other intracellular events. Conventional lithographic techniques using photoresists and micro-patterned molds provide a feasible way to fabricate the nanomaterials, however, it may be not a suitable methodology to produce biomedical-use materials due to the necessary irradiate process. To address this problem, we previously proposed a convenient method to pattern murine fibroblasts by engineering the physiochemical properties of free-standing nanosheet (Fujie et al., 2011). Since then, various kinds of surface-modified polymeric nanosheets have been developed as novel scaffolds for cell organization, cell delivery, and cell hierarchical construction (Fujie et al., 2013, 2015; Shi et al., 2014; Otomo et al., 2020). Microporous thin films (referred to as porous nanosheets) were previously introduced to induce cell construction and cell alignment (Suzuki et al., 2016). ECM proteins such as fibronectin, collagen and laminin penetrate through the micropores to stabilize cell hierarchies constructed between multi-layered porous nanosheets. Additionally, it has been reported that porous nanosheets facilitated long-term cell culture (up to 2 weeks) with more than 80% cell viability and oriented cell arrangement (Nishiwaki et al., 2019). Most recently, we reported for the first time that porous nanosheets prepared by a solvent etching method could support the adipose-tissue derived stem cell (ASC) spheroids for high-magnification imaging owing to its transparency (Suematsu et al., 2020). In this study, we further verified that free-standing poly(D,L-lactic acid) (PDLLA) porous nanosheet could improve the 3D spheroid cell viability and proliferation in comparison with the conventional suspension culture, demonstrating its potential to be applied as a novel 3D culture scaffold, which is cost-effective and easy-to-handle.

## Materials and Methods

### Materials

The polymers, solvents and reagents used in this study were purchased from the following suppliers: Poly(D,L-lactic acid) (PDLLA, Mw = 300,000–600,000) from Polysciences, Inc. (Warrington, PA); Poly(vinyl alcohol) (PVA, Mw = 13,000–23,000), ethyl acetate and cyclohexane from Kanto Chemical, Co., Inc. (Tokyo, Japan); Polystyrene (PS, Mw = ∼280,000) and bovine fibronectin from Sigma-Aldrich Co., LLC. (St. Louis, MO); poly (ethylene terephthalate) (PET) film (Lumirror 25T60) from Panac Co., Ltd. (Tokyo, Japan).

### Preparation of Porous Nanosheet

Using the approach proposed by Suzuki et al. (2016), the porous nanosheet were generated by Micro Gravure TM coater ML-120 (Yasui Seiki Co., Ltd., Kanagawa, Japan), a gravure coating method combining the roll-to-roll process. PVA solution (20 mg/mL) was coated first on the PET film to serve as a sacrificial layer, which could be dissolved and detach nanosheets from the PET substrate. From the polymer solution, the PET film runs through the coater, which rotates at 30 rpm, with a 1.3 m/min line speed over 7 meters. The solvent-coated film was high temperature air-dried (100°C) for about 5 min until the remaining solution has been evaporated. PDLLA and PS were then mixed in a 1:1 weight ratio and dissolved in ethyl acetate to make the polymer concentration of 20 mg/mL, which was optimized for cell culture purpose in terms of pore uniformity and diameters, porosity and thickness of nanosheets (Suzuki et al., 2016). This polymer solution was topped on PVA-coated film, and it went through the same coating process as the PVA layer except for a lower temperature (80°C) for drying. The obtained nanosheet was cut into 2 cm × 2 cm pieces, and then immersed into cyclohexane to selectively dissolve and remove PS regions by solvent etching. Without the PS regions, a random porous topography was produced on the remaining PDLLA nanosheet.

### Measurement of Thickness and Porous Diameter

Before the measurements, the substrate supported porous nanosheet was placed in deionized water to dissolve the PVA layer between the PET film and the porous nanosheet. Then the free-standing porous nanosheet was collected and reattached on a silicon wafer in deionized water in order to measure the thickness of porous nanosheet and porous diameter. The porous and non-porous nanosheets were examined with a profilometer (Dektak XT-S, Bruker BioSpin Co., Kanagawa, Japan), which uses two scarred scratches on the nanosheet attaching on a silicon wafer as the base to compare and obtain the relative height to represent nanosheet thickness. An atomic force microscope (AFM; VN-8000, Keyence Co., Ltd., Osaka, Japan) was applied to characterize the topographical surface. For the porous area, the ImageJ software package (U.S. National Institutes of Health, Bethesda, MD) was employed to measure the diameter using the phase-contrast images of porous nanosheets.

### Cell Culture

Murine adipose-derived stem cells (mASCs) (<10 passages; Cyagen Biosciences, Santa Clara, CA) were incubated at 37°C, 5% CO_2_ atmosphere in a cell culture media [Dulbecco’s Modified Eagle Medium: Nutrient Mixture F-12 (DMEM/F-12)] containing 10% (v/v) fetal bovine serum (FBS) and 1% (v/v) penicillin streptomycin. When the confluence reached 80%, cells were detached from the tissue-culture dish by using 0.25% (w/v) trypsin/0.1% (w/v) ethylenediaminetetraacetic acid (EDTA) after washing with Dulbecco’s phosphate-buffered saline (DPBS; Thermo Fisher Scientific, Inc., Carlsbad, CA). Murine embryonic stem cells (mESCs) were cultured in DMEM-knockout medium containing 15% FBS, 1% PS, 1% Glutamax, 1% NEAA, 0.1% β-mercaptoethanol, 0.2% leukemia inhibitory factor (LIF) and two inhibitors (PD0325901 and CHIR99021) at 37°C, 5% CO_2_ atmosphere.

### Spheroids Formation and Characterization

Collected stem cells (2 × 10^5^ cells) were seeded on the porous nanosheet, which had been fixed on a glass-based dish (thickness: 0.15–0.18 mm, 35 ø glass base dish, AGC Inc., Tokyo, Japan) with silicone elastomer: poly(dimethyl-siloxane) (PDMS). The same cell density was applied to non-adherent 12-well plate as well. The culture media would be changed every 2 days to keep cell survival. Cell aggregations were observed and the images were captured using an inverted microscope (IX-71; Olympus) from day 1 to 5 after cell seeding. According to the phase-contrast images, the diameter of cell aggregations was analyzed on ImageJ.

### Live/Dead Staining

After 72 h of cell seeding, Live/Dead viability/cytotoxicity test kit (Thermo Fisher Scientific) was used to observe the living and dead cells distribution within the spheroids. The kit includes two staining solutions, calcein AM targeting living cells and ethidium homodimer labeling dead cells. Calcein AM is hydrolyzed by esterase in living cells to become calcein, which retains in the cell cytoplasm and strongly emits green fluorescence; while ethidium homodimer only penetrates cells with disrupted plasma membrane and intercalates into DNA. Calcein AM and ethidium homodimer were mixed and diluted in PBS as instructed by the manual. After washing mASC spheroids collected from the nanosheet and the suspension culture media with PBS, 1 mL staining solution were added to each culture dish, followed by 15 min incubation at 37°C. Then, suspension cells were transferred into a glass-based dish prior to the observation under a confocal laser-scanning microscope (FV1000, Olympus Life Science). The green and red fluorescence intensity was separately analyzed by ImageJ and the ratio of living and dead cells in spheroids was compared between different groups. The corrected total cell fluorescence (CTCF) value was calculated using the formula (1) shown below:

(1) $$\begin{array}{c} \text{CTCF}=\mathrm{Integrated}\mathrm{density}-(\mathrm{area}\mathrm{of}\mathrm{the}\mathrm{selected}\mathrm{cells}\\ \;\times \mathrm{mean}\mathrm{fluorescence}\mathrm{of}\mathrm{background}\mathrm{readings}) \end{array}$$

### Cytoskeleton Staining

On day 3 after seeding, mASC spheroids were first fixed with 4% paraformaldehyde for 10 min and followed by treatment with 5% Trition X-100 (Wako Pure Chemical Industries, Osaka, Japan) in PBS for 15 min to disrupt the cell membrane after washing. In order to prevent background fluorescence, cells were treated with 10% bovine serum albumin (BSA, Sigma-Aldrich, United States) at room temperature for about 30 min for blocking of non-specific binding of antibodies. A dilution (1:500) of anti-Vinculin antibody (ab18058, Abcam, Cambridge, United Kingdom) was then used as the primary antibody to mark vinculin for overnight incubation at 4°C. After washing away the free anti-vinculin antibodies, a mixture of staining solution, containing Alexa Fluor 488-conjugated goat anti-mouse IgG (H + L) secondary antibody (A11001, Thermo Fisher Scientific), DAPI (4’,6-diamidino-2-phenylindole, Invitrogen^TM^ ) and Alexa Fluor 594 Phalloidin (Invitrogen^TM^ ) were added and incubated with cells at room temperature for at least 45 min. DAPI and Phalloidin were used to label the nuclei and filamentous actin (F-actin), respectively. The suspension spheroids were transferred into glass-based dish prior to the observation using a confocal microscope.

### Proliferation Assay

Spheroid cell proliferation assays were performed by using eBioscience^TM^ Cell Proliferation Dye eFluor^TM^ 670 (ThermoFisher). 1 × 10^6^ stem cells were counted and stained before seeding. The culture medium containing serum were first washed away with DPBS, and then cells were resuspended in 1mL DPBS complemented with 5 μL cell proliferation eFluor^TM^ 670 dye. After incubating and staining in dark at 37°C, the staining reaction was stopped by adding 4–5 mL cold FBS and incubating the cells on ice for 5 min. Cells were spun down (280 g, 5 min, 4°C) and washed once with culture medium. Then, 2 × 10^5^ cells were seeded in non-adherent 12-well plate as suspension culture or on a porous nanosheet to form spheroid. On day 3, the spheroids were harvested and dispersed into single cells with 100 μL trypsin/EDTA. After 5–8 min incubation at 37°C, the fluorescent intensity was monitored with a Cytomics FC500 flow cytometer (Beckman Coulter). The initial signal on day 0 was measured right after the staining process as the basis for the cell division calculation. The eFluor^TM^ 670 dye specifically binds to primary amines in cellular proteins and it would be dispensed evenly when the cell divides. According to the fading fluorescence, number of cell divisions and cell cycle duration were calculated using the following formula (2) and (3):

(2) $$\begin{array}{c} \mathrm{Number}\mathrm{of}\mathrm{cell}\mathrm{divisions}={\text{Log}}_{2}[\left(F_{0}-F_{\mathrm{unstained}}\right)/\\ \;\left(F_{\mathrm{samples}}-F_{\mathrm{unstained}}\right)] \end{array}$$

(3) $$\begin{array}{c} \mathrm{Cell}\mathrm{cycle}\mathrm{duration}\left(\text{hours}\right)=\mathrm{cell}\mathrm{culture}\mathrm{duration}\left(\text{hours}\right)/\\ \;\mathrm{number}\mathrm{of}\mathrm{cell}\mathrm{divisions} \end{array}$$

Where *F*_0_ and *F*_*samples*_ indicate the median fluorescence intensities of spheroid cells on day 0 and day 3, respectively; *F*_*unstained*_ indicates the background signal of unstained cells.

### Apoptosis Analysis

PE Annexin V Apoptosis Detection Kit I (BD Bioscience) was used to analyze the spheroid cell apoptosis cultured as suspension and on porous nanosheets. PE Annexin V detects the loss of membrane integrity accompanying the latest stage of cell death. It is often used in combination with 7-AAD, which labels dead or damaged cells without intact membranes, to identify viable cells (double negative), early apoptotic cells (PE annexin V positive, 7-AAD negative) and dead cells (double positive). The cells collected from both culture systems were washed with DPBS and treated with trypsin/EDTA in 37°C water-bath for 5–8 min to deconstruct the spheroids into single cells. Next, culture medium was added to stop the trypsinization, and the single cells were washed with DPBS. As the instruction describes, PE annexin V and 7-AAD were mixed and diluted in 1X Annexin V binding buffer, and samples were added to the staining solution. The cells were incubated and protected from light at room temperature for 15 min and then diluted with 1X Annexin V binding buffer before analyzing with the Cytomics FC500 Flow cytometer. For the positive control, cells were exposed under UV-light for 3–4 h.

### Statistical Analysis

All statistical analyses were performed using one-way ANOVA by StatsPlus software. *P*-value significance was determined as <0.005 (^∗∗∗^), <0.01 (^∗∗^), and <0.05 (^∗^).

## Results

### Preparation and Characterization of Porous Nanosheet

**FIGURE 1:**
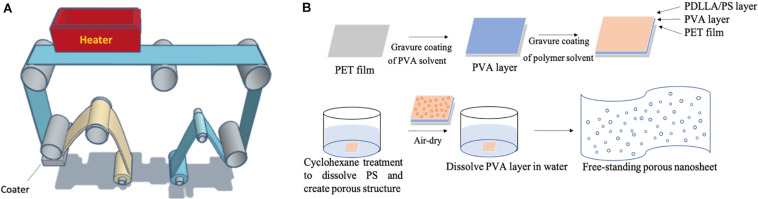
**(A)** Image of the gravure-coating method combining with roll-to-roll process to prepare nanosheet. **(B)** Schematic illustration of free-standing porous nanosheet preparation.

Porous nanosheets were integrated from two immiscible polymers, PS and PDLLA (1:1 in weight) via a gravure-coating method (Figure 1A), followed by a solvent etching process with cyclohexane to remove the phase-separated PS regions (Figure 1B).

**FIGURE 2:**
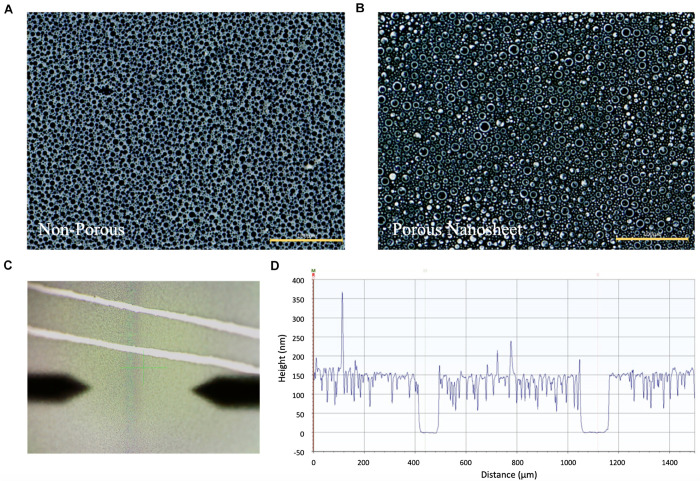
The phase-contrast images of **(A)** non-porous nanosheet and **(B)** porous nanosheet (scale bar: 100 μm). Measurement of the thickness of nanosheet. **(C)** Image of nanosheet obtained from the profilometer. Two parallel scratches were gently made to expose the silicon substrate. The surface topography was profiled to obtain the height difference to represent film thickness **(D)**.

As indicated by the phase-contrast images in Figures 2A,B, the porous nanosheet obtained after cyclohexane treatment showed bubble-like, shiny and porous topography, whereas the PS regions remaining in the non-porous nanosheet block the light from optical microscopy and exhibited as little black circles. The thickness of nanosheet was calculated from the relative height of nanosheet surface to the substrate surface using a stylus profilometer (Figures 2C,D). The resulting porous nanosheets were 168 nm in thickness and had an average porous area of 6.6 μm^2^ with a porosity of 0.887, whereas the non-porous nanosheet was 228 nm in thickness (Table 1).

**TABLE 1:** Characterization of non-porous nanosheet and porous nanosheet.

Sample	Thickness (nm)	Porous area (μm^2^)	Porosity
Non-porous nanosheet	228	N/A	N/A
Porous nanosheet	168	6.6	0.887

### Spheroid Formation on Porous Nanosheet

The edges of porous nanosheets were fixed on a glass-based dish with a silicone elastomer; poly-dimethyl-siloxane (PDMS), to proceed *in vitro* experiments. To observe how porous nanosheets affect the morphology of mASCs, phase-contrast images were captured every day after cell seeding. Representative images of day 1, 3, and 5 were chosen to demonstrate the differences in spheroid size and morphology among mASCs seeded on porous nanosheets and those in non-adherent dishes as suspension culture (Fang and Eglen, 2017; Lv et al., 2017).

**FIGURE 3:**
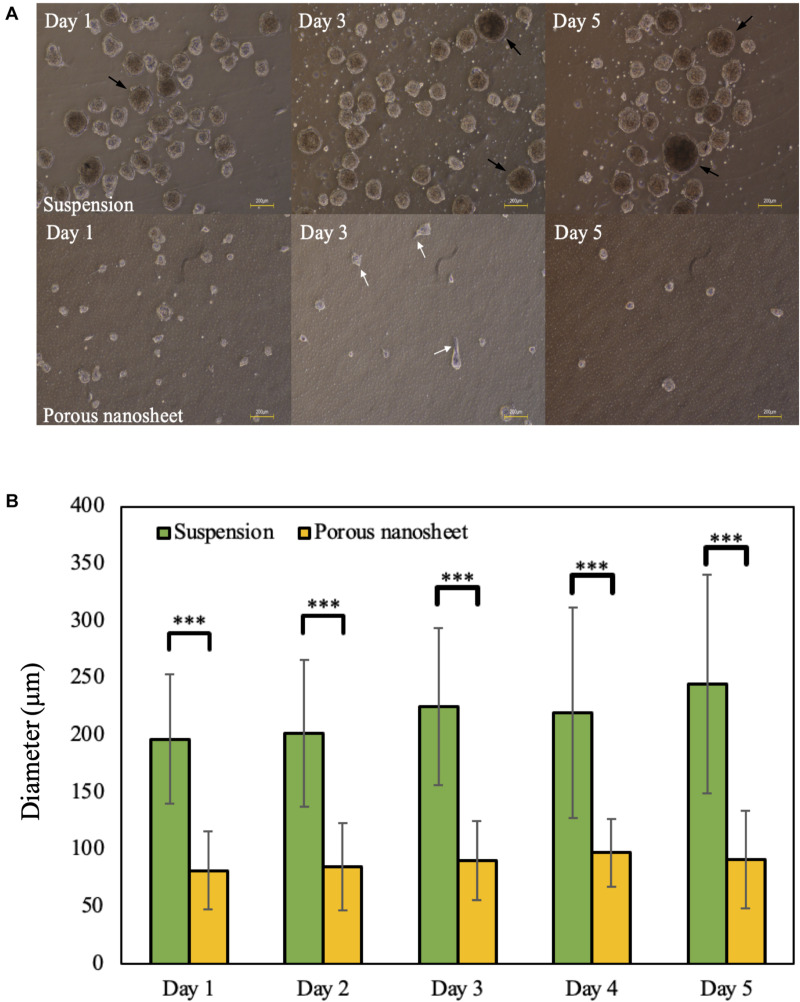
**(A)** The morphology of mASC spheroids cultured in suspension (top) and on porous nanosheets (bottom) on days 1, 3, and 5. Spheroids showed the attachment on porous nanosheet on day 3 as indicated by white arrows and representative spheroid clusters formed in suspesnsion culture were indicated by black arrows. Scale bar: 200 μm. **(B)** The average diameter of spheroids formed in suspension and on porous nanosheets. Data show the mean ± SD (*n* = 5, ^∗∗∗^*p* < 0.005).

Within the first 24 h, mASCs aggregated as spheroids both in suspension culture and on the porous nanosheet (Figure 3A). Spheroids in suspension culture tended to cluster together (represented by black arrows) as compared to spheroids on the porous nanosheet, which were evenly distributed. On day l, spheroids had grown to an average diameter of 225 μm in suspension culture and 90 μm for spheroids on the porous nanosheet (Figure 3B). Furthermore, the spheroids cultured on the porous nanosheet started developing as a tethered state to the porous surface as indicated by the white arrows. Until day 5, the spheroids in suspension culture had grown to an average diameter of 250 μm in irregular shapes, whereas spheroid size on the porous nanosheet remained below 100 μm in average while keeping a spherical shape. Taken together, the porous nanosheets promoted a stable generation of mASC spheroids with a better homogeneity in size and shape.

### Live/Dead Staining of Spheroids

**FIGURE 4:**
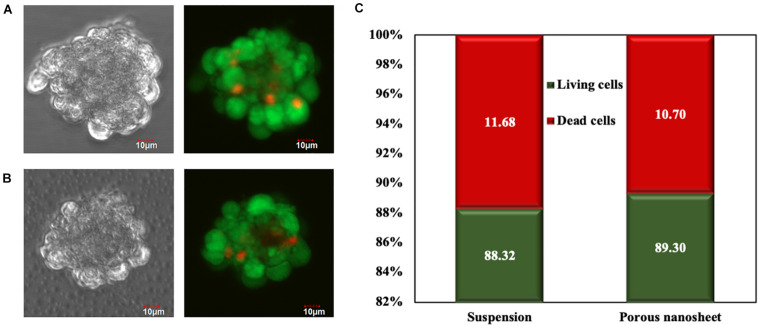
Live/Dead cell staining of mASC spheroids of a similar size formed in suspension **(A)** and on the porous nanosheet **(B)** on day 3. Scale bar: 10 μm. **(C)** The percentage of living and dead cells in spheroids was calculated by the corrected total cell fluorescence (CTCF) intensity.

To evaluate cell viability within the spheroids, Live/Dead cell staining was conducted on day 3. Confocal images of spheroids on the porous nanosheet were captured through the porous nanosheet owing to its ultra-thin and transparent characteristics, which does not interfere with focusing the spheroids (Suematsu et al., 2020). It was found that dead cells mostly presented in the core region of spheroids regardless of culture environment (Figures 4A,B). Then, the fluorescence intensity was analyzed to confirm the Live/Dead cell ratio within the spheroids (Figure 4C). After eliminating the background intensity, the correlated total cell fluorescence (CTCF) indicates that the proportion of living and dead cells was approximately 9:1 in both culture systems. It is suggested that cell viability of spheroids was not affected by the external stress from the two different culture environments and they supported cell viability to a similar extent. Note that spheroids of similar sizes were analyzed for comparison.

### Cytoskeleton Observation

**FIGURE 5:**
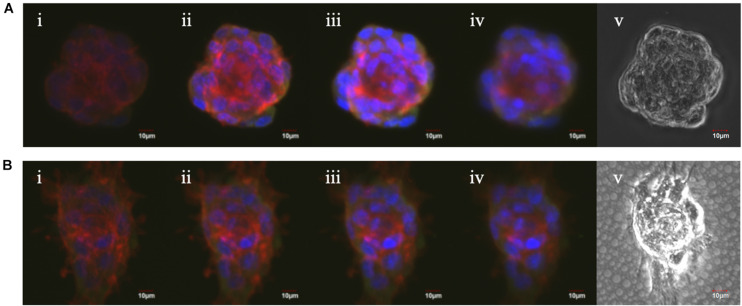
The Z-stack fluorescent images of mASC spheroids in suspension culture **(A)** and on the porous nanosheet **(B)** from the bottom (i) to the top (iv) view and the phase-contrast images (v). Red: Alexa Fluor 594 Phalloidin; green: Anti-Vinculin antibody; blue: DAPI.

In order to confirm spheroid attachments on the porous nanosheets, vinculin and f-actin were visualized. Vinculin, a globular protein associated with cell adhesion and lamellipodia formation, interacts with f-actin and is identified as a mechano-transducer when developing focal adhesion (Tolbert et al., 2013). When cells anchor down on the substrate and spread like a monolayer culture, f-actin expands radially and colocalizes with vinculin at the pointed end of actin filaments (Noriega and Subramanian, 2011). A confocal laser scanning microscope was used to capture z-stack images of the spheroids obtained from suspension culture or a porous nanosheet, starting on the bottom part of spheroids (i) and ending at the apex of spheroids (iv) (Figure 5). F-actin filaments in spheroids from the porous nanosheet noticeably expanded two-dimensionally and adhered on the nanosheet illustrated by the strongest intensity at the bottom half of the sphere. In contrast, focal adhesions were not observed for spheroids cultured in suspension and the f-actin fluorescence was mainly apparent in the central part and on the edge of the spheroid. It is also interesting to find that the cell (nuclei) orientation is different. The spheroid cells in suspension culture appeared to be circumferentially oriented and tightly compressed, whereas those generated on the porous nanosheet did not show uniformed orientation at the bottom part and only the cells at the apex part tended to be circumferentially oriented. Additionally, the colocalization of f-actin and vinculin was clearly observed in a circumferential manner surrounding the suspension spheroids. In comparison, when seeded on the porous nanosheet, f-actin seemed to be evenly distributed and appeared with relatively weak signals only at the cell-junctions within the spheroids.

### Proliferation Assay

**FIGURE 6:**
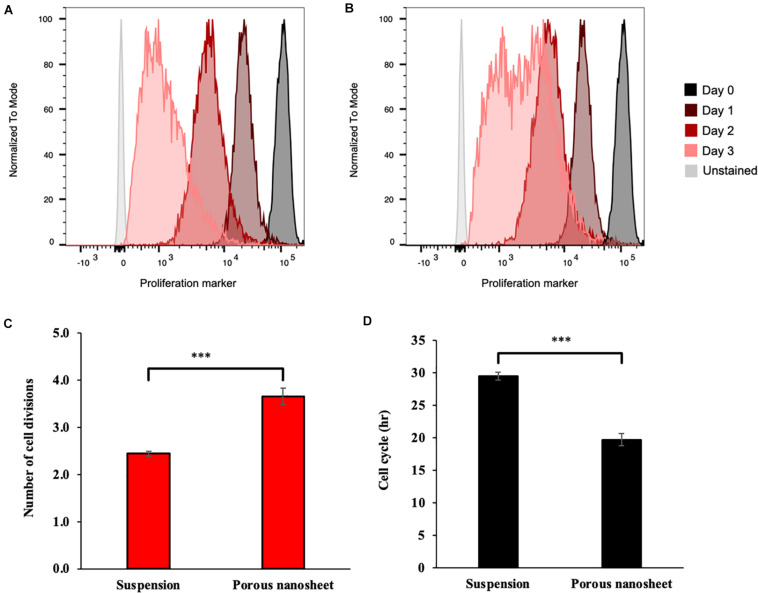
Representative flow cytometry histograms of the proliferation assay, showing fluorescence reduction of cells from spheroids cultured on porous nanosheet **(A)** and in suspension **(B)** from day 1 to 3. Number of cell divisions **(C)** and average cell cycle duration **(D)** in hours (h) of mASC spheroids were calculated according to the loss of fluorescence on day 3 relative to initial fluorescence on day 0. Data show the mean ± SD (*n* = 3, ^∗∗∗^*p* < 0.005).

As mentioned above, the spheroids in both culture systems continued to develop during the culturing period, which might be due to the aggregation and fusion of spheroids or cell proliferation within the spheroid itself. Thus, a cell proliferation dye that binds to cellular proteins was used to analyze cell divisions overtime by flow cytometry, assuming that upon every cell division, the dye fluorescence intensity decreases to the half. As shown in Figures 6A,B, the cell division profiles of two culture systems were remarkably different on day 3. In suspension culture, there was a portion of spheroid cells that have decelerated or terminated the division; whereas most of those seeded on the porous nanosheets continued to proliferate from day 2 to 3. It was estimated that the mASC of spheroids in suspension culture divided about 2.5 times while those adhered on the porous nanosheet divided approximately 4 times after 3 days of incubation (Figure 6C). From the number of cell divisions in this time frame, the cell cycle duration could be roughly gauged. It took nearly 30 h for the spheroid cells in suspension culture to divide once, whereas it took about 20 h for those seeded on the porous nanosheets (Figure 6D). The mESC spheroids constructed on the porous nanosheets also showed a similar trend, with shorter, though not statistically significant, estimated cell cycle durations than the suspension culture (Supplementary Figure 1). Overall, these results showed that the porous nanosheet could sustain the spheroids in a prosperous state with higher proliferation rates than those in the suspension culture.

### Apoptosis Analysis

The Live/Dead staining allowed the visualization of cell viability in the spheroids of similar sizes; however, it is difficult to give an overall evaluation including all the spheroids. Therefore, cell apoptosis assay was performed by using PE annexin V and 7-AAD double staining in order to comprehensively analyze the cell death without size discrimination and investigate the progress of apoptosis.

**FIGURE 7:**
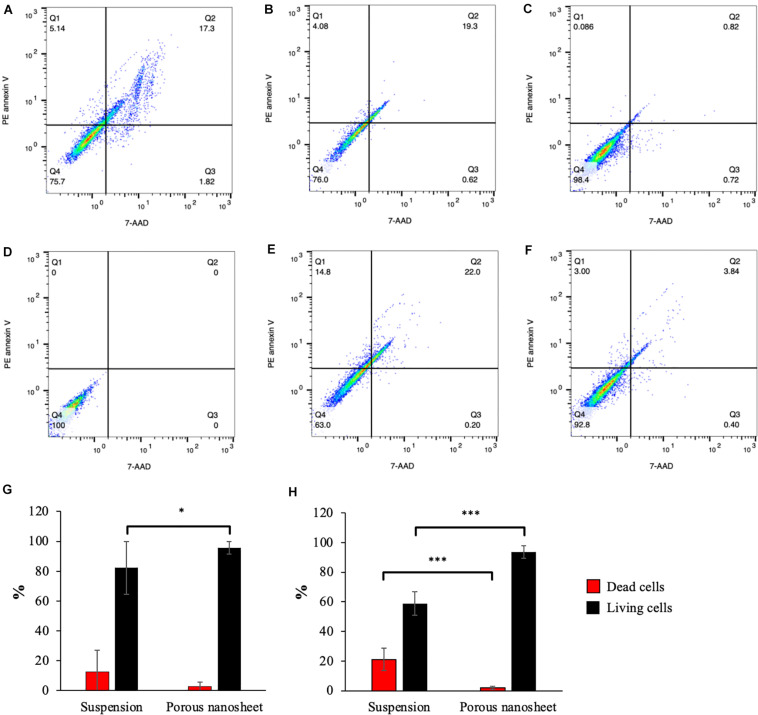
Apoptosis analyses of cells from spheroids cultured on porous nanosheets or in suspension. Representative PE annexin V apoptosis scatter plots of positive control treated with UV-light **(A)**, spheroids in suspension **(B,E)** and on porous nanosheet **(C,F)** harvested on day3 **(B,C)** and day 5 **(E,F)**, and unstained negative control **(D)**. Percentage of dead cells (Q2) and living cells (Q4) in mASC spheroids on day 3 **(G)** and day 5 **(H)**. Data show the mean ± SD (*n* = 3, ^∗^*p* < 0.05, ^∗∗∗^*p* < 0.005).

The cells were harvested from mASC spheroids on day 3 and 5, dissociated by trypsinization of spheroids, labeled and analyzed by flow cytometry. PE annexin V and 7-AAD double negative cells were gated in Q4 on the scatter plots (Figures 7A–F) to represent living cells. Early apoptotic cells were defined in Q1 gates (PE annexin V positive, 7-AAD negative) while double positive cells were identified in Q2 gates as dead cells. On day 3, the mASC spheroids in suspension contained almost 20% of dead cells whereas less than 3% of dead cells existed on the porous nanosheet (Figure 7G). On day 5, the population of living cells in the suspension spheroids shrank to 58%; in comparison, 93% of the spheroid cells on porous nanosheet remained alive (Figure 7H), the difference of which was remarkably significant. Notably, suspension culture also resulted in a small portion of early apoptotic cells on day 5, implying the progress of apoptosis. Similarly, it was confirmed with mESC spheroids that the porous nanosheet enhanced the cell viability with over 50% of living cells on day 5 in contrast to mESCs suspension spheroids, which contained about 40% of living cells (Supplementary Figures 2, 3). With the support of porous nanosheet, spheroids could be better preserved from apoptosis/necrosis than suspension culture and possibly used for long-term culture for comprehensive studies.

## Discussion

PDLLA is a hydrophobic polymer with high biocompatibility and biodegradability. Due to its amorphous nature, it exhibits a lower mechanical strength and a faster degradation rate than the crystalline poly(L-lactic acid) (PLLA), making it more preferrable to engineer soft tissue scaffold (Ulery et al., 2011). In contrast, PLLA is widely applied for bone tissue engineering after blending with hydrophilic polymers such as poly (glycolic acid) (PGA) to accelerate the degradation (Shuai et al., 2021). We previously reported that PDLLA porous nanosheets could induce the generation of mASC spheroids owing to their porous topology and hydrophobic property (water contact angle: 74°), which provides a weakly adhesive surface to allow the cell attachment as well as migration (Suematsu et al., 2020). In this study, we further demonstrated that this 3D culture scaffold had several advantages over the conventional suspension culture, as it better promoted cell viability and cell proliferation, as well as a better homogeneity of spheroid’s size and shape.

3D culture provides a close-to-*in vivo* microenvironment to study cell-cell interaction and spatial effects. However, due to the lack of angiogenesis, radial gradients of nutrients, gasses and growth factors are generated, reducing the viability of spheroid cells toward the core by decreased accessibility to nutrients and stimuli (Mehta et al., 2012; Cui et al., 2017). Therefore, the diameter of spheroids is thought to be a key factor in determining the cell viability. To verify whether the porous nanosheet imposes additional disturbance to affect the homeostasis, the spheroids of similar sizes were used for comparable quantifications, i.e., in the Live/Dead staining and cytoskeleton observation.

The mechanical properties within spheroids depend on the spatial position. In the core region, cells are progressively compressed in the radical direction, while the cells in the peripheral area further bear an increased circumferential stress, forming a contractile outer shell with more nuclei to drive the spheroid compaction (Lee et al., 2019). At an early stage of spheroid formation, cell-cell contact is relatively loose, and cells are either circumferentially, radially, or not oriented within the spheroids. Following the increase of spheroid size, structural anisotropy is generated, transducing compression forces toward the core which imposes mechanical anisotropy as a response of the cells. Such mechanical stress would lead to remodeling and changes in cell orientations, with the circumferentially oriented cells dominating the edge of spheroids, while the radially oriented cells occupying the central region (Dolega et al., 2017; Lee et al., 2019). Surprisingly, the cell orientation and the distribution of vinculin and f-actin were different in the two culture systems, indicating that the spheroids were bearing different kinds of mechanical stress and undergoing different fates. The porous nanosheet controlled the diameter of mASC spheroid by offering the porous scaffold for spheroids to cling on, which reduced cell aggregations. Therefore, the porous nanosheet might provide a culture system that favors the early stage of spheroid formation through proper size control.

In general, both the porous nanosheet and the non-adherent dish showed 3D spheroid construction capability. The suspended mASC spheroids had aggregated rapidly in the first 24 h, yet they developed into uneven sizes during the 5 days incubation. From Live/Dead cell staining, it was revealed that cell death started from the central region of the spheroids of both suspension and porous nanosheet culture systems, which may be caused by the shortage of oxygen and nutrition supplies; however, there was no distinguishable difference when analyzing by similar sizes. Living and dead cell ratios were almost 9:1 from both culture systems (Figure 4), in this regard, 20% of dead cells in suspended spheroids analyzed by flow cytometry (Figure 7) might have resulted from relatively large spheroids. Given the fact that the porous nanosheet provides limited area of attachment, stem cells could loosely and sparsely adhere to it to form spheroids instead of a monolayer (Suematsu et al., 2020). Meanwhile, the spheroid size would be controlled by lowering the opportunity of spheroid fusion or aggregation in terms of collision frequency and movement speed of spheroids. The crawling-like f-actin fluorescence depicts the spheroid attachments on the porous nanosheets, verifying the semi-adhesive property of porous nanosheet, which is crucial to control the spheroid size. As a result, the spheroids anchoring on the porous nanosheet reduce interaction with each other so that preventing further development into larger spheroids as those floating in the suspension. The different culture environments contribute to the spheroid formation with different sizes, and the size difference affects spheroid biological performances including cell proliferation and apoptosis. Stem cells are characterized by a high potential of proliferation throughout the lifetime of organisms, and a high proliferation rate is thought to be crucial in maintaining human embryonic stem cell identity (Ruiz et al., 2011). It is no doubt that a sustained stem cell proliferation is a favorable feature for its application in tissue engineering and regeneration (Zhao et al., 2013; Zhou et al., 2016). Overall, mASC spheroids cultured on the porous nanosheet proliferated faster and survived longer when comparing to those forming in the suspension, implying a higher potential of PDLLA porous nanosheet in maintaining the stem cell functions and applicability for tissue engineering. However, further investigations on the pluripotency and differentiation potential of spheroid stem cells are necessary for a comprehensive evaluation. In addition to mASCs, porous nanosheets are also applicable to generate and sustain mESC spheroids although the improvement was not statistically significant. Note that advanced coating such as Matrigel is commonly used to support mESC culture *in vitro*, its lack on the porous nanosheet was likely to compromise the spheroid formation. Therefore, a combination of Matrigel and the porous nanosheet is expected to further improve the preservation of mESC spheroids.

In conclusion, porous nanosheets have shown its improvement in 3D cell constructions by providing efficient semi-attachment, which appears to limit the spheroid size. With preserved spheroids, porous nanosheets could be a promising scaffold for advanced long-term spheroid culture and close-to-*in vivo* study.

## Data Availability Statement

The original contributions presented in the study are included in the article/Supplementary Material, further inquiries can be directed to the corresponding author/s.

## Author Contributions

YT, LT-F, and SW conducted the experiments and performed data analysis. YT wrote the first draft of the manuscript. TL guided the experiments, organized the manuscript, and performed data analysis. WK and ST conceived and supervised the study. All authors contributed to manuscript revision.

## Conflict of Interest

ST was an inventor of patent (PCT/JP2013/056823) of porous nanosheets and collaborating with Nanotheta Co., Ltd., which is holding the patent. The remaining authors declare that the research was conducted in the absence of any commercial or financial relationships that could be construed as a potential conflict of interest.
