# Supplementary material for: Ultra-Thin Porous PDLLA Films Promote Generation, Maintenance, and Viability of Stem Cell Spheroids
Source: Front Bioeng Biotechnol. 2021 Jun 14;9:674384. doi: 10.3389/fbioe.2021.674384 (PMC8236593; doi:10.3389/fbioe.2021.674384)
Supplement: Supplementary file 1 [file Data_Sheet_1.PDF]

## Supplementary

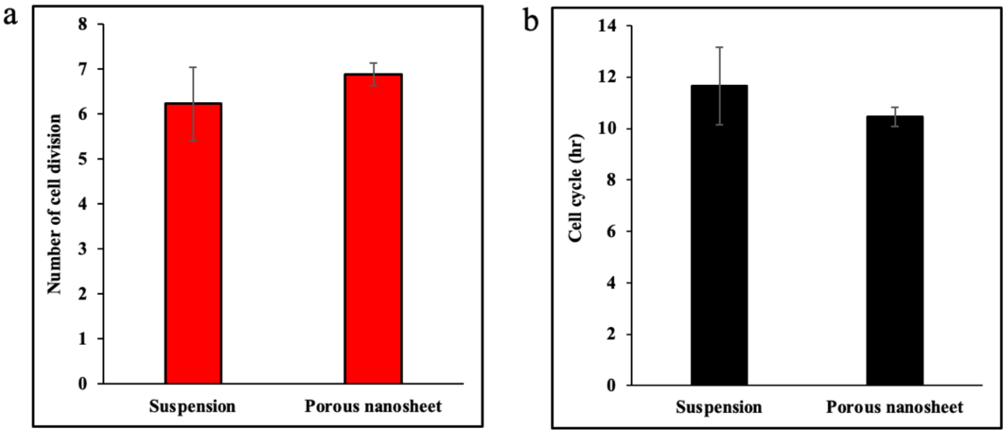


**sFigure 1.** (a) Number of cell divisions and (b) cell cycle duration in hours (hr) of mESC spheroids. According to the loss of fluorescence intensity on day 3 relative to the initial fluorescence intensity at day 0, the number of cell divisions was calculated and used to further estimate the cell cycle duration in hours (hr). Data show the mean ± S.D. (n=3)

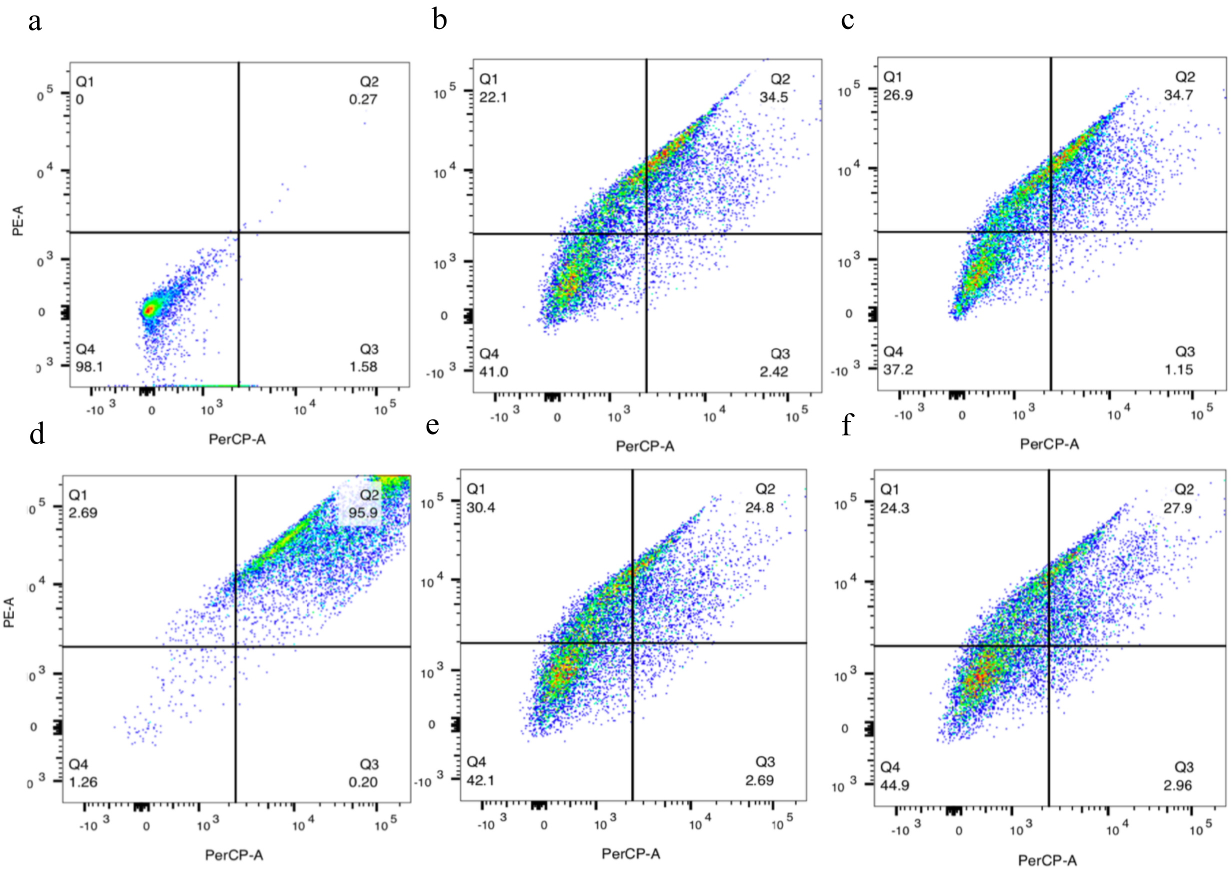


**sFigure 2.** Representative PE annexin V scatter plots (x-axis: 7-AAD; y-axis: PE annexin V) of mESC spheroid cells. (a) Unstained negative control; (b, e) suspension spheroids on day 3 and day 5; (c, f) porous nanosheet spheroids on day 3 and day 5; (d) positive control treated with UV irradiation.

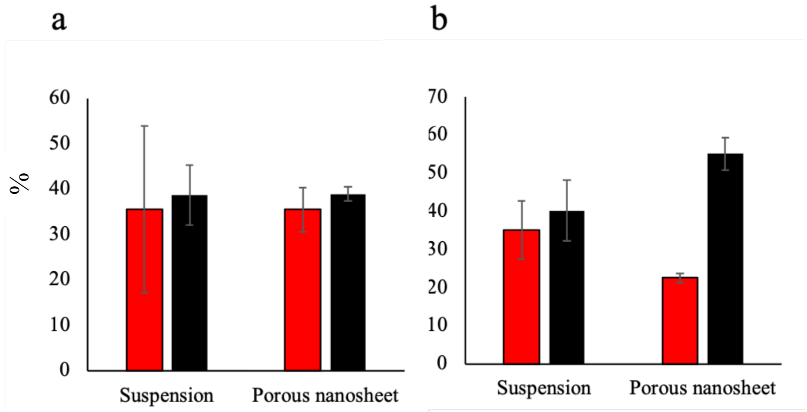


**sFigure 3.** Percentage of dead cells (red bars) and living cells (black bars) in mESC spheroids on day 3 (a) and day 5 (b). Data show the mean ± S.D. (n=3)
